# Costs of insensitive acetylcholinesterase insecticide resistance for the malaria vector *Anopheles gambiae *homozygous for the G119S mutation

**DOI:** 10.1186/1475-2875-9-12

**Published:** 2010-01-13

**Authors:** Luc Djogbénou, Valérie Noel, Philip Agnew

**Affiliations:** 1 Institut Régional de Santé Publique/Université d’Abomey-Calavi, 01 BP 918 Cotonou, Bénin; 2 Lutte contre les Insectes Nuisibles (LIN IRD UR016), 911 Avenue Agropolis, Montpellier 34394, France; 3 Génétique et Evolution des Maladies Infectieuses (GEMI CNRS-IRD UMR 2724), 911 Avenue Agropolis, Montpellier 34394, France

## Abstract

**Background:**

The G119S mutation responsible for insensitive acetylcholinesterase resistance to organophosphate and carbamate insecticides has recently been reported from natural populations of *Anopheles gambiae *in West Africa. These reports suggest there are costs of resistance associated with this mutation for *An. gambiae*, especially for homozygous individuals, and these costs could be influential in determining the frequency of carbamate resistance in these populations.

**Methods:**

Life-history traits of the AcerKis and Kisumu strains of *An. gambiae *were compared following the manipulation of larval food availability in three separate experiments conducted in an insecticide-free laboratory environment. These two strains share the same genetic background, but differ in being homozygous for the presence or absence of the G119S mutation at the *ace-1 *locus, respectively.

**Results:**

Pupae of the resistant strain were significantly more likely to die during pupation than those of the susceptible strain. Ages at pupation were significantly earlier for the resistant strain and their dry starved weights were significantly lighter; this difference in weight remained when the two strains were matched for ages at pupation.

**Conclusions:**

The main cost of resistance found for *An. gambiae *mosquitoes homozygous for the G119S mutation was that they were significantly more likely to die during pupation than their susceptible counterparts, and they did so across a range of larval food conditions. Comparing the frequency of G119S in fourth instar larvae and adults emerging from the same populations would provide a way to test whether this cost of resistance is being expressed in natural populations of *An. gambiae *and influencing the dynamics of this resistance mutation.

## Background

The presence of the G119S mutation in the *ace-1 *gene [1,2] has recently been reported from populations of *Anopheles gambiae *in the West African countries of the Ivory Coast, Benin and Burkina Faso [3-9]. This mutation confers resistance to organophosphate (OP) and carbamate (CX) insecticides by reducing the ability of these compounds to inhibit acetylcholinesterase (AChE) in nerve synapses [1,2]. Its presence poses a potential problem for the control of these malaria vectors in the region.

The G119S mutation is responsible for AChE insensitivity in several species of mosquito [10], and it has been extensively studied in natural populations of *Culex pipiens *in the south of France [11,12]. These studies found an important factor determining the frequency of resistant mosquitoes are the costs of resistance they experience in areas untreated with OP or CX insecticides [13]. The source of these costs can be traced to the negative effects the mutation has on a variety of traits related to the life-history, physiology and reproductive success of resistant mosquitoes (Table 1[14-23]). These studies have also found the costs of resistance for the G119S mutation are expressed in a partially-dominant manner and that selection is stronger against homozygous individuals in untreated areas than it is against heterozygotes [11].

**Table 1:** Summary of traits associated with fitness costs for the G119S mutation for *Culex pipiens/C. quinquefasciatus *mosquitoes in insecticide-free environments

**Trait**	**Reference**
	
*field studies*	
longer developmental time	[14]
adult size (shorter wing length)	[14]
reduced survival of over-wintering females	[15,16]
overall fitness	[13,17]
	
*laboratory studies*	
reduced male mating success	[18]
reduced probability of adult emergence	[19-21]
reduced metabolic reserves at emergence	[19]
smaller adult size (tibia length)	[20]
increased risk of predation	[22]
reduced female fecundity	[20]
higher *Wolbachia *load	[23]

Far less is currently known concerning the costs of resistance for the G119S mutation when expressed in *Anopheles *mosquitoes. However such information is essential to understand the dynamics of resistance mechanisms and can be useful helping design strategies for the control of resistant populations in areas where malaria is prevalent.

It has been predicted that the costs of the G119S mutation should be similar for *An. gambiae *and *Culex pipiens *as the strength of resistance and biochemical properties of the modified AChE enzyme are almost identical in the two species [24]. It is already known that its strength of resistance is expressed in a partially-dominant manner in *An. gambiae *[5], as it is in *Culex *mosquitoes. Field data from the Ivory Coast and Burkina Faso indicate the costs of resistance are also expressed in a partially-dominant manner, as there is a strong deficit in the frequency of homozygous individuals compared to that expected from Hardy-Weinberg equilibrium proportions [4].

In this study, the costs of resistance for *An. gambiae *s.s. with the G119S mutation were investigated in an insecticide-free laboratory environment. The expression of resistance mutations and the life-history traits they influence are often sensitive to the environmental conditions (genetic, biotic, abiotic) in which they are assessed. These potentially confounding effects were minimized by (i) comparing a sensitive and resistant strain of *An. gambiae *that share a common and sensitive genetic background, but differ in the presence or absence of the G119S mutation, (ii) studying the mutation in its homozygous state, so as to avoid the problem of environmentally-sensitive variation in the strength of dominance expressed in the heterozygous state [25], (iii) rearing larvae in the absence of density-dependent competition, and (iv) performing replicate experiments in controlled laboratory conditions.

## Methods

### The G119S mutation

The *ace-1 *gene in mosquitoes codes for acetylcholinesterase enzyme (AChE1) in the nervous system [26]. This enzyme hydrolyses the neurotransmitter acetylcholine (ACh) bound to receptors on post-synaptic membranes of neural synapses and thus terminates transmission. Most OP and CX insecticides target and irreversibly bind to AChE. This causes ACh to accumulate in the synaptic cleft, leading to paralysis of the insect and its death.

Almost all cases of resistance to OP and CX involving an insensitive AChE1 are due to a single mutation in the *ace-1 *gene [1,2,26]. This is a GGC-to-AGC point mutation leading to a glycine-to-serine amino-acid substitution at position 119, or G119S, following the *Torpedo *nomenclature; it has also been referred to as *ace-1R *or *ace-1*^*R*^. Models of enzyme structure locate this substitution in the region surrounding the target of many OP and CX insecticides. Its presence can explain the reduced ability of insecticides to bind to their target and consequently their reduced insecticidal activity [24]. The modified enzyme also shows a reduced binding affinity to its normal substrate, ACh [27].

### Mosquito strains

Two strains of *An. gambiae *s.s. were used in this study. Kisumu is a reference strain susceptible to all insecticides. It was originally isolated in the Kisumu region of western Kenya in the early 1950s and has been maintained in the laboratory since then [28]. The other strain was AcerKis, which is homozygous for the G119S mutation and resistant to both OP and CX insecticides [5]. This strain has the same genetic background as the Kisumu strain due to 19 generations of back-crossing and selection (with propoxur) between Kisumu and resistant *An. gambiae *caught in the Bobo-Dioulasso region of Burkina Faso in 2002 [5]. Both strains are of the 'S' molecular form of *An. gambiae *s.s. and are equally sensitive to several concentrations of DDT and deltamethrin, suggesting resistance alleles for P450 (metabolic resistance) are not present [5]. Diagnostic PCR tests did not detect the presence of the *kdr *mutation conferring resistance to DDT and pyrethroid insecticides (L. Djogbénou, pers. comm.).

### Experimental protocol

This study involved three experiments in which life history traits of the Kisumu and AcerKis strains were recorded after they developed in insecticide-free environments varying in larval food availability. The three experiments were separated in time and involved mosquitoes from different generations of the two strains. In each case, the parental populations of both strains had experienced the same laboratory conditions and had been reared with the same protocol for several generations. Adult females blood-fed on restrained rabbits and the oviposition of the two strains was synchronized to generate matching cohorts of larvae.

Each experiment started with a total of 960 first instar larvae. In each case, 480 first instar larvae from each strain were transferred to their own individual *Drosophila *tube (diam. 25 mm × 95 mm) containing 5 ml of mineral water (Eau de Source, Carrefour, France). Rearing larvae individually avoids the results being influenced by density-dependent interactions among larvae [29]. This approach is particularly relevant for this type of study as any differences in the strength of density-dependent competition within populations of either susceptible or resistant larvae would confound the observed results, e.g., see [30] for how a parasitic infection influences the strength of competition within and between infected and uninfected larvae of *Aedes aegypti*.

Larvae were fed daily according to their food treatment (see below) until they either pupated or died. In the event of pupation, the date was recorded and the tube sealed with a foam bung to prevent the emerging adult from escaping. In the event of adult emergence, the date was recorded. No food was provided to adults, but they had access to the water in their tubes, thus forcing them to survive by metabolising nutritional reserves accumulated during larval development. The day of adult death was recorded and individual cadavers were transferred to a numbered 1.5 ml plastic vial and stored at -20°C until further treatment. The day of death of individuals dying as either larvae or pupae was also recorded before they were transferred to individually numbered vials and stored at -20°C.

Once all the adults from within an experiment had been collected they were dried at 60°C for a minimum of 12 h before their dry starved weight was measured to an accuracy of ± 1 μg with a Mettler Toledo MX5 balance (Mettler-Toledo GmbH, Greifensee, Switzerland).

### Larval food treatments

Each experiment involved four larval food treatments, but experiments differed in the amount of food provided. In each case larvae were fed daily with a known quantity of fish food (Tetramin BabyMin, Tetra Gmbh, Melle, Germany) dissolved in 200 μl of water. The treatments used were either 0.075, 0.100, 0.125, 0.150, or 0.175 mg of food per larva per day. The weights were chosen to provide a range of food conditions varying in their favourability for larval growth [19]. The 0.100, 0.125, 0.150 and 0.175 mg treatments were used in the first experiment, while the second and third experiments involved the 0.075, 0.100, 0.125 and 0.150 mg treatments.

In each experiment the 960 larvae were arranged in 24 racks containing 40 vials. Within each rack there were four rows of 10 tubes, two rows contained Kisumu larvae and two contained AcerKis larvae. All the larvae within a particular rack received the same food treatment.

Eight pots of food were prepared each day, two for each food treatment. These pots contained enough food for 200 larvae dissolved in 40 ml of water. Each pot was used to feed the larvae in three racks. The identity of the racks to be fed from a particular pot was chosen randomly at the beginning of an experiment and maintained throughout the experiment.

The first two experiments took place in an insect room maintained at 27°C (± 1°C) and > 65% relative humidity. The racks in these experiments were physically arranged on a single surface in six blocks of four racks, with one rack per food treatment. For the third experiment, racks were distributed among three incubators maintained at 27°C, with two racks from each of the four food treatments per incubator. Racks within an incubator were moved at random on a daily basis to reduce positional effects.

### Statistical analyses

There were three separate experiments and each experiment involved a split-plot design. The whole plots were the food treatments (four per experiment). Each whole plot was replicated twice in each experiment, corresponding with two separate preparations of each food treatment. The split-plots involved the effect of strain within each whole plot. For adult traits the effect of an individual's sex was added as a further split-plot within the effects of food treatment and strain.

Fully factorial analyses of variance (ANOVA) were used to test the fixed effects of food treatment, strain and sex (where appropriate). Experiment and food replicate within food treatments were treated as random effects. Analyses were performed with JMP version 5.1.2 [31] or R version 2.7.0 [32].

For adult traits, the R code used with the *nlme *package [33] was:

model <- lme (y ~ food*strain*sex, random = ~ 1|experiment/food/food replicate/strain/sex)

## Results

### Pre-adult mortality

There was variation among experiments for mortality within the first 48 h of larvae being placed in their tubes. However this variation was not associated with either strain or larval food treatments (Table 2a). In the three experiments, 9%, 1% and 41% of the Kisumu larvae died within 48 h, while mortality of the AcerKis larvae was, 36%, 1% and 4%, respectively.

**Table 2:** Summary results from analyses of variance on pre-adult mortality

**(a) Early larval mortality (< 48 h)**^**1**^				
**Source**	**nDF**	**dDF**	***F***	**p**
Food (F)	4	5	0.215	0.919
Strain (St)	1	19	1.024	0.324
F.St	4	19	1.744	0.182
Error		19		
				
**(b) Larval mortality (> 48 h to pupation)**				
**Source**	**nDF**	**dDF**	***F***	**p**
Food (F)	4	5	1.068	0.460
Strain (St)	1	19	1.432	0.246
F.St	4	19	1.316	0.300
Error		19		
				
**(c) Mortality during pupation**^**1**^				
**Source**	**nDF**	**dDF**	***F***	**p**
Food (F)	4	5	41.569	**< 0.001**
Strain (St)	1	19	9.698	**0.006**
F.St	4	19	1.190	0.347
Error		19		

nDF = nominator degrees of freedom

dDF = denominator degrees of freedom

^1 ^Arcsine square-root transformed data

Once the initial 48 h had passed, the majority of the remaining individuals completed their larval development and pupated (58%, 88% and 55%, for the three experiments respectively). The probability of mortality in the period following the initial 48 h and until pupation was unrelated to either strain or food treatment (Table 2b).

**Figure 1:**
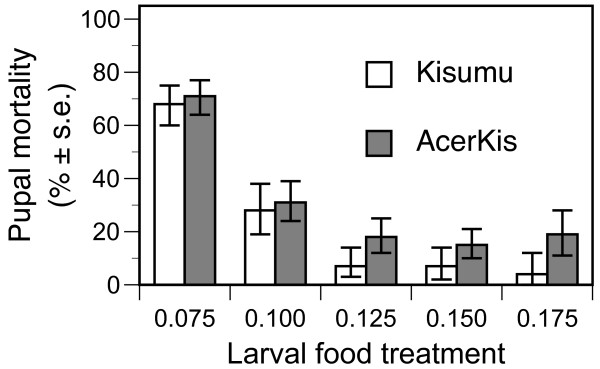
**Percentage mortality during pupation of the susceptible Kisumu (open columns) and resistant AcerKis (full columns) strains of *Anopheles gambiae *when provided with diets varying in larval food availability**. Mean values and standard errors are least square mean estimates from the split-plot model described in the text.

Individuals of the resistant AcerKis strain were more likely to die during pupation than those of Kisumu (Table 2c, Figure 1). Pupal mortality tended to increase as larval food availability decreased, but there was no interaction between strain and food availability (Table 2c, Figure 1).

### Adult traits

**Figure 2:**
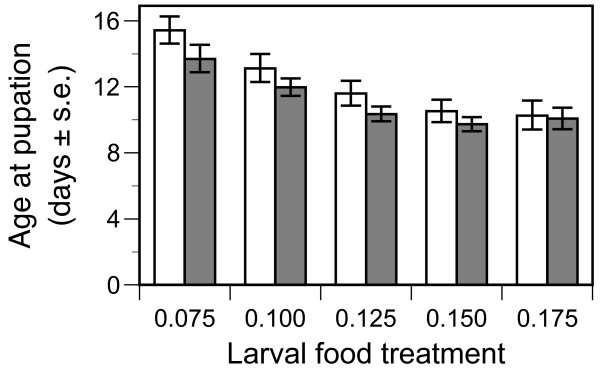
**Age at pupation of the susceptible Kisumu (open columns) and resistant AcerKis (full columns) strains of *Anopheles gambiae *when provided with diets varying in larval food availability**. Mean values and standard errors are least square mean estimates from the split-plot model described in the text.

Individuals successfully emerging as adults pupated earlier as larval food availability increased and on average those of the AcerKis strain pupated roughly half a day earlier than those of Kisumu (Table 3a, Figure 2). Males pupated earlier than females (Table 3a).

**Table 3:** Summary results from analyses of variance on mosquito life-history traits

**(a) Log_**10 **_(Age at pupation)**				
**Source**	**nDF**	**dDF**	***F***	**p**
Food (F)	4	5	49.303	**< 0.001**
Strain (St)	1	19	22.377	**< 0.001**
Sex (Se)	1	37	34.617	**< 0.001**
F.St	4	19	0.775	0.555
F.Se	4	37	0.321	0.862
St.Se	1	37	0.308	0.583
F.St.Se	4	37	0.878	0.487
Error		1147		
				
**(b) Dry adult weight**				
**Source**	**nDF**	**dDF**	***F***	**p**
Food (F)	4	5	93.459	**< 0.001**
Strain (St)	1	18	23.994	**< 0.001**
Sex (Se)	1	37	150.028	**< 0.001**
F.St	4	18	0.434	0.782
F.Se	4	37	2.230	0.085
St.Se	1	37	0.100	0.753
F.St.Se	4	37	1.172	0.339
Error		1093		
				
**(c) Adult longevity**				
**Source**	**nDF**	**dDF**	***F***	**p**
Food (F)	4	5	1.065	0.461
Strain (St)	1	19	0.579	0.456
Sex (Se)	1	37	130.299	**< 0.001**
F.St	4	19	0.477	0.752
F.Se	4	37	0.486	0.746
St.Se	1	37	1.066	0.309
F.St.Se	4	37	0.889	0.480
Error		1131		

nDF = nominator degrees of freedom

dDF = denominator degrees of freedom

**Figure 3:**
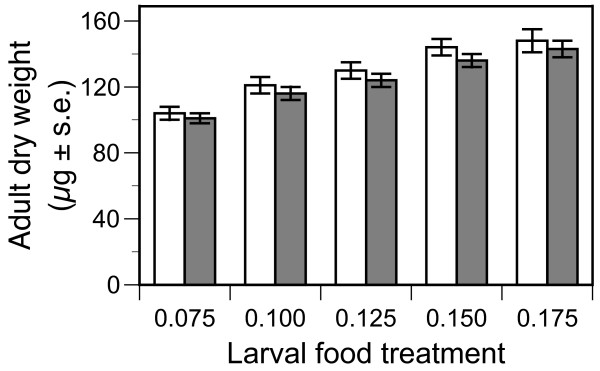
**Dry starved weight of adult mosquitoes of the susceptible Kisumu (open columns) and resistant AcerKis (full columns) from of *Anopheles gambiae *when provided with diets varying in larval food availability**. Mean values and standard errors are least square mean estimates from the split-plot model described in the text.

The dry starved weight of adults was measured as an index of their size. Adults of the AcerKis strain were on average ~ 5% lighter than their Kisumu counterparts (Table 3b, Figure 3). Both strains became heavier as larval food availability increased (Table 3b, Figure 3). Females were generally heavier than males (Table 3b).

**Figure 4:**
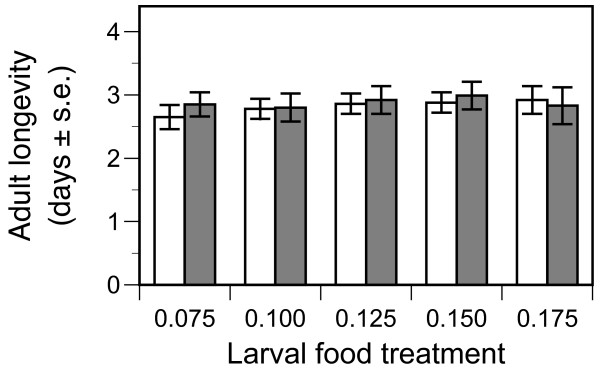
**Adult longevity (age at death - age at pupation) of the susceptible Kisumu (open columns) and resistant AcerKis (full columns) strains of *Anopheles gambiae *when provided with diets varying in larval food availability**. Mean values and standard errors are least square mean estimates from the split-plot model described in the text.

The period between pupation and the death of unfed adults was not influenced by either strain or larval food treatment (Table 3c, Figure 4). However, females tended to survive approximately half a day longer than males (Table 3c).

## Discussion

The aim of this study was to assess the costs of resistance associated with the homozygous state of the G119S mutation based on life-history traits of *An. gambiae *in an insecticide-free laboratory environment.

No significant differences in larval mortality were found for the two strains (Table 2a, b). However during pupation the mortality of the resistant strain was greater than that of the sensitive strain (Table 2c). When data from each experiment and food treatment were combined, ~ 31% of all AcerKis pupae died during pupation (265/868), compared with 20% for the Kisumu strain (162/802). This represents a substantial cost for resistant individuals as it directly decreases the proportion of individuals able to contribute to the next generation. Furthermore, the difference between the two strains was seen across food treatments and included the higher food conditions where pupal mortality was lowest (Figure 1). This indicates this cost is likely to be expressed even in environments favourable for mosquito development.

Previous laboratory studies have found increased levels of pre-adult mortality for *C. quinquefasciatus *mosquitoes bearing the G119S mutation [19-21]. However in these studies larval and pupal mortality were not reported separately. A re-inspection of the data in [19] found no significant difference in mortality for pupae of the insensitive acetylcholinesterase strain and the sensitive strain (P. Agnew, pers. comm.), whereas substantial pupal mortality was observed in [21] for the insensitive acetylcholinesterase strains (M. Weill, pers. comm.).

AChE has been identified as having a role during insect development [34]. For example, its activity has been reported as maximal in the pre-pupal stage of *Drosophila melanogaster *[35] and at adult emergence for *Apis mellifera *queens [36]. Hence, the pupal mortality observed for the AcerKis strain may be due to its modified AChE causing disruption to developmental processes during metamorphosis. Further data are required to verify if this is a general effect extending to other mosquitoes and/or in which conditions it is most likely to be expressed.

Comparing the frequency of the G119S mutation in fourth instar larvae and adults emerging from the same sites would provide a means to test whether pupal mortality is a cost experienced in natural mosquito populations. Furthermore, such data should be refined to examine the relative frequencies of homozygous and heterozygous individuals in the respective populations. This would provide useful information as to the relative strength of selection acting on mosquitoes with one or two copies of the allele. Based on what is already known from *Culex *mosquitoes [11], it can be anticipated that there will be less pupal mortality for heterozygous individuals and their mortality will vary relatively more from site to site than for homozygous individuals as the dominance of the mutation is environmentally variable [25].

The fitness of resistant and sensitive mosquitoes not only depends on their respective chances of reaching adulthood but also on the reproductive success they achieve as adults. Two important life-history traits influencing reproductive success are age and size at maturity. In these experiments AcerKis individuals tended to reach pupation earlier than those of the Kisumu strain (Table 3a), but they also emerged as smaller adults (Table 3b). The possibility that AcerKis adults were smaller because they pupated earlier can be discounted as the difference in the weights of the strains remained when individuals from each strain were matched for ages at pupation (mean [AcerKis - Kisumu] = -5.438 μg, *t *= 5.895, d.f. = 125, *p *< 0.001; comparison of mean dry weights of each strain when matched for age at pupation, experiment, food treatment, replicate food group within experiment, and sex).

Earlier ages at pupation indicate potential fitness benefits for the resistant strain. For example, shorter developmental times could lead to reduced generation times and reduced risk of larval mortality due to predation or their aquatic environment drying out. The reproductive success of resistant adult males could also benefit from earlier emergence if it increases their encounter rate with previously unmated females. In contrast, the smaller adult size of resistant individuals indicates potential fitness costs for resistant mosquitoes. For example, smaller females are likely to be less fecund and to experience greater risks of mortality while blood-feeding due to the need to feed more frequently. The influence of adult size on the reproductive success of *Anopheles *males is less clear, varying considerably among studies [37-39].

Previous studies involving *Culex *mosquitoes have produced mixed results concerning age and size at maturity. Later ages at pupation and smaller adult size were found for resistant individuals in a field study involving *C. pipiens *[14]. An individual's resistance status did not influence either trait in a laboratory study involving *Culex quinquefasciatus *[19], but in different experimental conditions resistant individuals were found to be smaller [20]. Age and size at maturity were not reported, but the mating success of resistant male *C. quinquefasciatus *was less than that of sensitive males when both compete for females [18].

The above studies do not show a clear pattern for the effects of the G119S mutation on the age and size at maturity of mosquitoes. They also suggest any effects it has on these traits are likely to be obscured by variation in environmental conditions experienced during larval growth.

The G119S mutation in its homozygous state was not found to influence the time that adult mosquitoes could survive by metabolising reserves accumulated during larval development (Table 3c, Figure 4). This suggests metabolic costs are not particularly associated with this form of insecticide resistance, whereas such costs are important in other forms of insecticide resistance, e.g., esterase overproduction [40].

## Conclusions

Costs associated with the G119S mutation in its homozygous state were found for the fitness of *An. gambiae *mosquitoes in an insecticide-free laboratory environment. The main cost was due to greater mortality of resistant individuals during pupation relative to their sensitive counterparts. Furthermore, this mortality occurred in the absence of density-dependent competition among larvae or pupae and was expressed across a range of treatments varying in larval food availability, including those most favourable for growth. This indicates a general cost of resistance for *An. gambiae *that was not influenced by the biotic or abiotic conditions encountered in these experiments. There was also evidence for costs to adult fitness as resistant individuals were smaller than sensitive adults, however these costs could be offset by their shorter developmental times observed for resistant individuals.

These results support preliminary field data finding fitness costs associated with being homozygous for the G119S mutation in natural populations of *An. gambiae *in West Africa [4]. If pupal mortality during pupation is a factor in these costs it could be detected by testing field populations for the frequency of the mutation in fourth instar larvae relative to that in adults emerging from the same populations.

## Competing interests

The authors declare that they have no competing interests.

## Authors' contributions

LD and PA designed the experiments, analysed the data, and wrote the manuscript. LD, VN and PA participated in the experiments and data collection. All authors read and approved the final manuscript.
